# Experimental and Modeling Analysis of Mechanical Response of Composite Electrodes in Lithium Batteries

**DOI:** 10.3390/molecules29143316

**Published:** 2024-07-14

**Authors:** Zheru Shen, Zhiyao Jin, Yaolong He, Dawei Li

**Affiliations:** 1 School of Mechanical Engineering, University of Shanghai for Science and Technology, Shanghai 200093, China; zhe_ru@163.com (Z.S.);; 2 School of Mechanics and Engineering Science, Shanghai University, Shanghai 200093, China

**Keywords:** mechanical response, composite electrodes, lithium batteries, in situ bending deformation measurement

## Abstract

The mechanical response is one of the main factors that influence the capacity and number of cycles of lithium batteries, which hinder its wide application. Therefore, it is crucial to perform an in-depth investigation of the electro-chemo-mechanical coupling performance and work mechanism of battery electrodes during the electrochemical reaction process. Usually, graphite is the main active material used in commercially used batteries, while silicon is gaining worldwide attention because of its large energy density. Here, graphite and silicon composite electrodes were prepared to obtain the electro-chemo-mechanical response during electrochemical cycling by an in situ bending deformation measurement. The findings indicate that the composite electrodes could induce a large bending deformation, with an increase in the state of charge (C-rate). And, with an increase in the C-rate, the deformation degree of the silicon composite electrode increases, while that of the graphite composite electrode decreases due to the hardening properties of the graphite particles. In addition, increasing the thickness ratio could induce an increase in the peak stress for both composite electrodes. This work gives a detailed analysis of the mechanical properties of composite electrodes and finds the working mechanism of the C-rate and thickness ratio, which can supply suggestions for the development of high-performance batteries.

## 1. Introduction

The reliability and longevity of lithium batteries have become critical for meeting the great increase in electric vehicles and renewable energy sources [1,2,3,4]. It is of the utmost urgency to develop next-generation lithium batteries with large energy densities, long cycle lives and stable performance under multiple conditions [5,6,7]. Basically, the insertion and extraction of lithium ions in an electrode can induce a significant volume change in the active particles, which can further cause significant stresses [8,9,10]. At a high C-rate, mechanical degradation plays an important and dominant role in the electrochemical performance of a cell, which causes large volume changes, leading to reduced Coulombic efficiency, increased voltage polarization and safety risks. These mechanical responses during electrochemical cycling can lead to the severe detachment of the active material, electrode damage, delamination and so on [11,12,13,14,15]. As a result, it is necessary to conduct an in-depth investigation to obtain a detailed understanding of the working mechanism of battery electrodes, which is crucial for developing the next generation of batteries.

Several experimental studies have been conducted to investigate the influence of the mechanical degradation of battery electrodes. Li et al. [16] developed an in situ measurement system to explore the influence of different coating densities on the coupled electro-chemo-mechanical performance of silicon electrodes, and the results showed the mechanical parameters were positively correlated with the calendering degree. Yu et al. [17] measured the deformation process of a silicon carbide composite electrode. The results showed that it increased during the charging process, and decreased during the discharging process. Leah et al. [18] employed a multi-beam optical stress sensor (MOSS) system to measure the irreversible changes in LR-NMC films. Also, this method was used to detect the stress changes in silicon electrodes with different binders during electrochemical cycling. The silicon electrodes could induce irreversible shape changes during the reaction process. Jones et al. [19] investigated irreversible capacity loss caused by structural change through digital image correlation (DIC). These results could supply basic experimental data for a comprehensive understanding of the stresses generated by composite electrodes. Therefore, further in-depth studies in conjunction with simulations are required.

Modeling and computational methods have also been employed to quantitatively analyze the stress changes due to the mechanical deformation of electrodes. Allen et al. [20] constructed a continuum-level damage model that studied the mechanical damage to lithium batteries during the first cycling. Zhang et al. [21] built a mechanical model and found that larger particle sizes and current densities increased the diffusion-induced stresses on spherical particles [10,22]. Song et al. [23] conducted a theoretical investigation into the effect of the mechanical properties. Their findings indicated that compressive stresses on the electrode surface hindered the embedding of Li ions. Cui et al. [24] developed a new stress-dependent chemical potential to investigate the distribution of diffusion-induced stress. Liu et al. [25] conducted a quantitative evaluation of the Li transport kinetics affected by stress. The above simulation analyses provide a good direction for this study, but there is a lack of experimental verification of the conclusions obtained from these simulations. Furthermore, there is a lack of understanding regarding the mechanism of how C-rates affect the stress evolution of composite electrodes.

Therefore, in order to address the research gap in the combined experimental and simulation approaches to studying the mechanical response of composite electrodes, this work combined experiments and simulations to investigate the effect of charge rates and thickness ratios on the electro-chemo-mechanical coupling performance of battery electrodes. Here, two kinds of composite electrodes were prepared, graphite and silicon. Then, a model cell was used to investigate the bending deformation process of the bilayer electrodes. The bending curvatures of the different electrodes were obtained by changing the charging rates and thickness ratios, respectively. Then, a physical model was established, and the lithium ion concentration, stress and curvature of the electrodes were analyzed. The simulation results provide an in-depth understanding of the mechanical response of the composite electrodes, which can be used to improve the efficiency and durability of the battery.

## 2. Results

**Figure 1:**
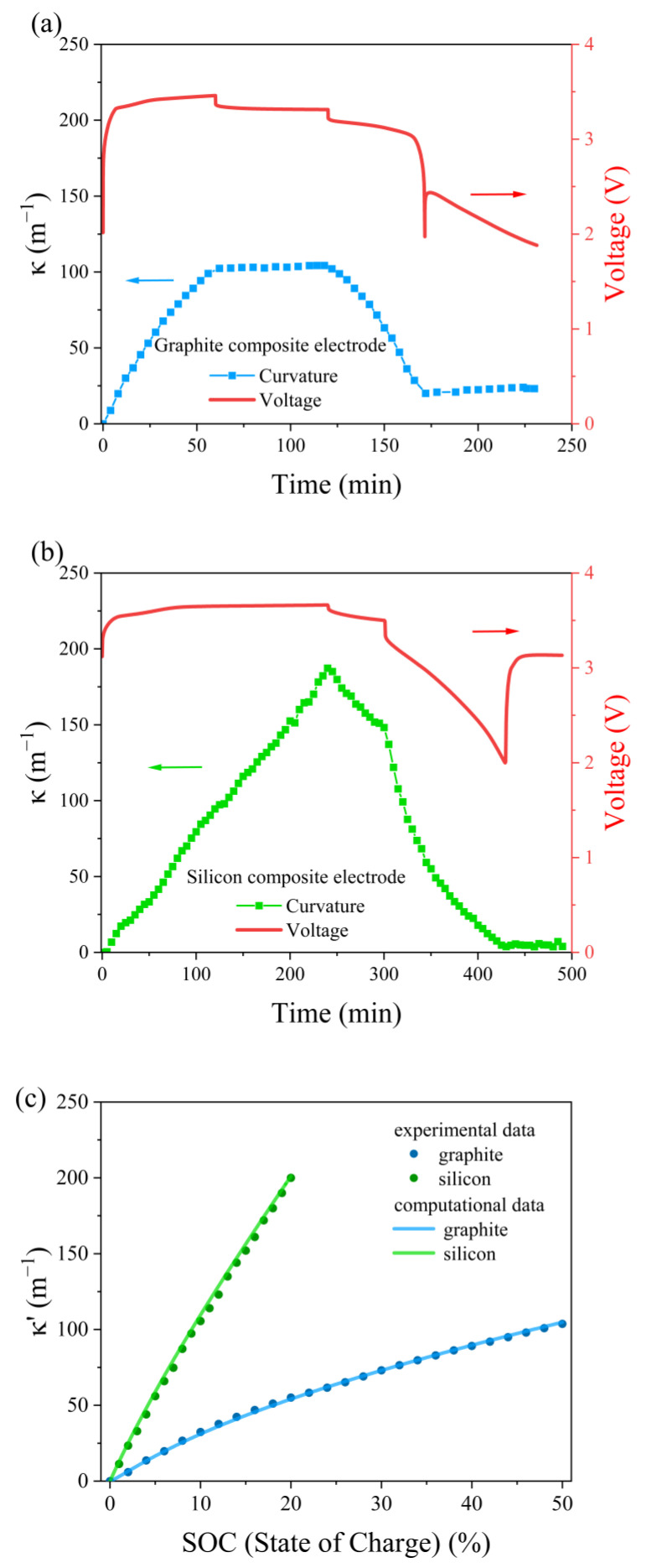
Voltage and curvature of (**a**) composite graphite electrode, (**b**) composite silicon electrode and (**c**) experimental and simulated normalized curvature variation in composite electrodes during lithiation.

Figure 1a,b illustrates the voltages and corresponding curvatures during the electrochemical lithiation and delithiation cycles for the composite graphite electrode at magnification and the composite silicon electrode. The 60 min open-circuit relaxation period evened out the distribution of the lithium ions’ concentration and eliminated the effect on the next cycle. The curvature was observed to increase with an increasing C-rate, due to asymmetric diffusion and the copper foil’s restriction. This indicates that the curvature is dependent on the concentration of lithium ions.

Figure 1c demonstrates that the curvature obtained from the simulation aligns closely with the curvature observed during the experiment, except for the fact that the graphite electrode deviates a bit at a low C-rate, which may be caused by the limited interfacial transport of lithium ions from the composite graphite electrode during the experiments [26]. This indicates that the parameters employed in the computational analysis align with the properties of the composite electrodes in the experiment.

**Figure 2:**
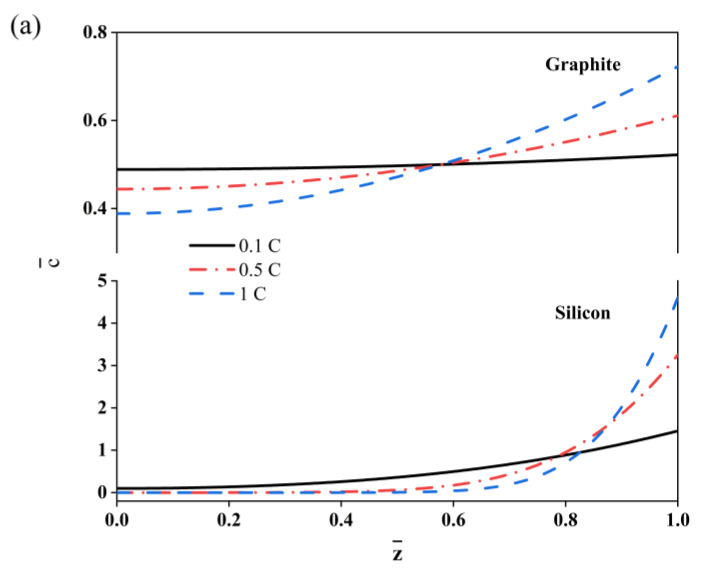

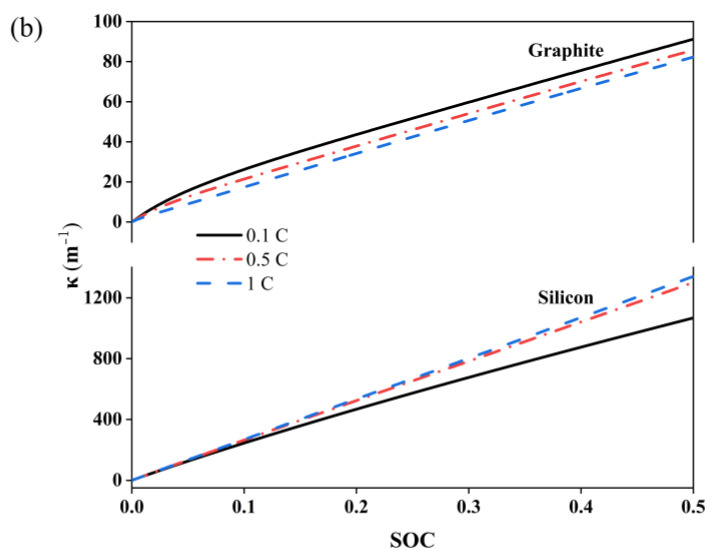
(**a**) Simulated dimensionless lithium concentration and bending curvature. (**b**) Simulated evolution of the bilayer electrodes during electrochemical cycling.

Given that the charge rate can greatly affect the electrochemical response of the composite electrodes, three charge rates were selected for the further analysis of the silicon and graphite composite electrodes, i.e., 0.1C, 0.5C and 1C, respectively. Here, the thickness ratio of the active layer and current collector ($h_{c}$/$h_{a}$) was set at 1/6, while the SOC was 50%. The diffusion of the dimensionless li ion concentration along the *z*-axis is illustrated in Figure 2a. It is obvious that the lithium ion concentration in the outer surface of the active layer is higher than it is at the interface for both the graphite and silicon electrodes. An increase in the C-rate can induce a large distribution gradient, and the lithium concentration is relatively small at the interface of the active layer and copper foil. Here, the lithium concentration of the silicon electrode at the interface is quite small due to its low diffusion coefficient. The C-rate can also affect the deformation process of the bilayer electrodes, as shown in Figure 2b. The curvature decreases with a larger C-rate for the graphite electrode, while it increases for the silicon electrode. This is mainly affected by the modulus change in the active materials. Usually, a graphite electrode hardens with the insertion of lithium ions [27], while a silicon electrode softens.

**Figure 3:**
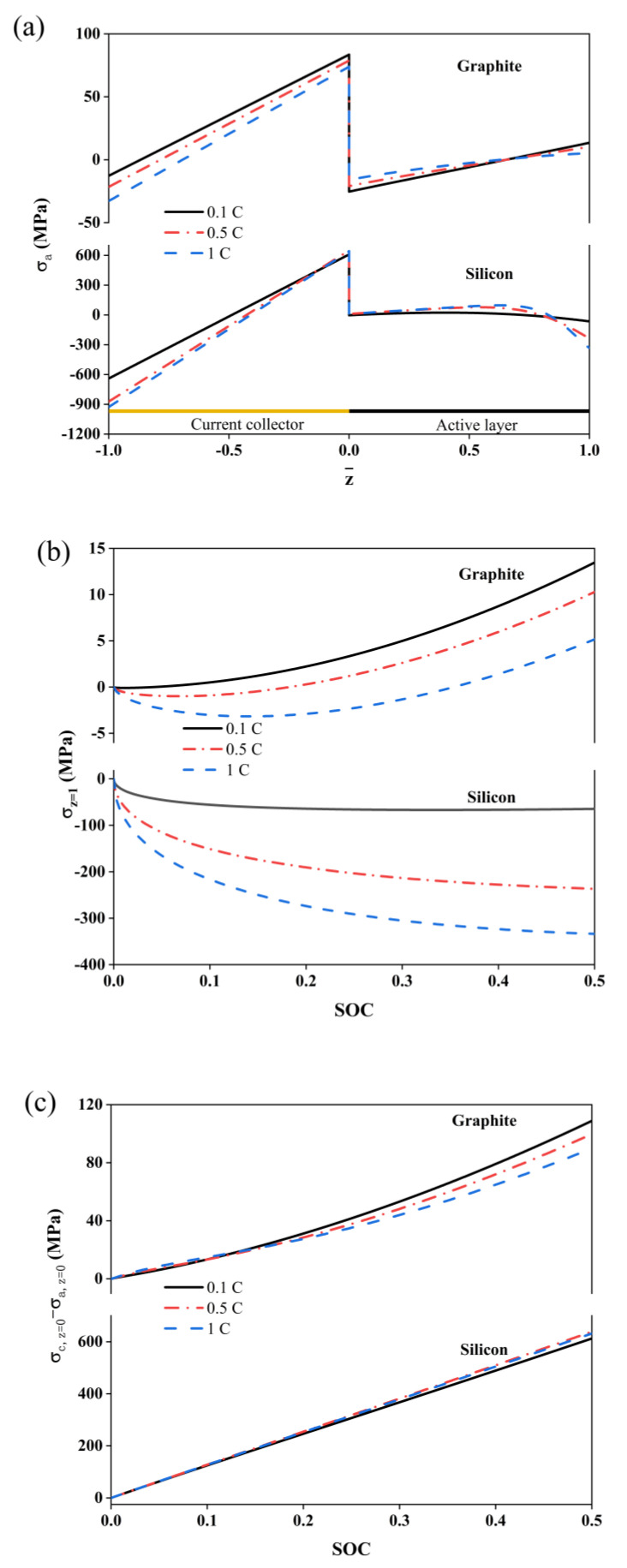
Simulated stresses in monolayer composite electrodes at different multiplicities. (**a**) Stress distribution, (**b**) surface pressure of active layer, and (**c**) differential stress at the active layer–collector interface.

The stress changes in the composite electrodes at different C-rates are illustrated in Figure 3. The current collectors in the composites undergo a tension state at the interface, which can restrict the expansion of the active layer, as shown in Figure 3a. In the active layer of the graphite composite electrode, the compressive stress at the interface is converted to tension because of the domination of the diffusion-induced stresses in this region. In addition, the bending stresses are dominated and changed to tensile stresses at the outer face. For the silicon composite electrode, the stresses are compressive due to the larger lithium ion concentration polarization at the outer face.

The stress of the outer surface and interface are depicted in Figure 3b,c. For both types of electrodes, the stresses increase with an increase in the charge rate. However, there is a difference; the graphite exhibits tensile stresses while the silicon exhibits compressive stresses. Moreover, the stress of the silicon electrode is approximately 100 times larger that of the graphite electrode, which is attributed to the large volume expansion of the silicon particles. At the interface between the active layer and the collector, the *D*-value of the stresses of the graphite composite electrode decreases with an increasing charge rate. An increase in the charge rate can lead to the decrease in lithium ions concentration at the interface, resulting in a decrease in the diffusion-induced stresses. However, the effect of the charge rate on the stress *D*-value for the silicon composite electrode is relatively small. Since the value of the magnitude of the stress in the silicon electrode is considerably larger than that in the graphite, it is obvious that the stress difference in the silicon is much larger than that in the graphite.

**Figure 4:**
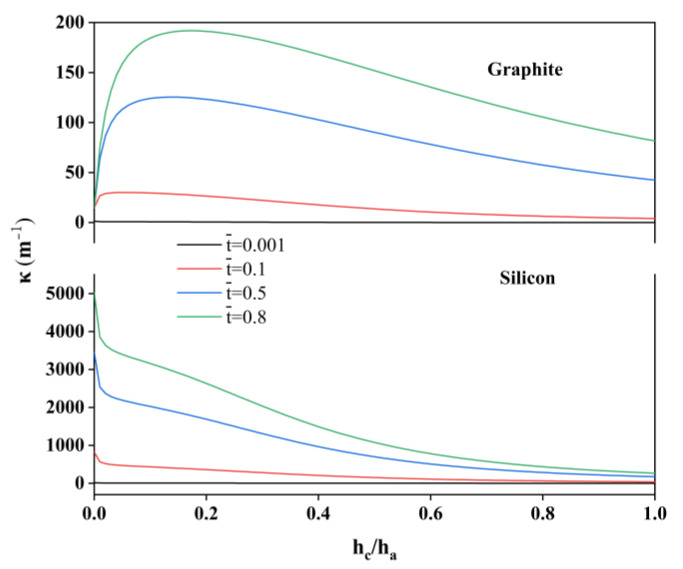
The simulated curvature variation for different thickness ratios of the bilayer electrodes.

The influence of the thickness ratio on the mechanical response of the electrodes is illustrated in Figure 4. Here, the thickness of the active layer is set at 40 µm, while the charge rate is 0.5 C. For the graphite and silicon, the dimensionless lithium ion concentration increases with the C-rate, and exhibits higher values at the active layer surface. Figure 4 illustrates the impact of the thickness ratio ($h_{c}/h_{a}$) on the curvature of the single-layer composite electrodes. For the graphite, when the current collector is quite thin, the deformation of the active layer is completely determined by the embedded lithium, resulting in a modest curvature. If the collector is loaded a certain thickness, the curvature could increase due to the increased confinement in the regions adjacent to the collector and active layer. As the thickness of the collector increases, the overall curvature of the graphite composite electrode decreases, as the collector has a higher modulus bending stiffness. However, for the silicon composite electrode, the smaller diffusion coefficient results in a lower concentration of lithium ions at the interface of the collector and the active layer and, therefore, the thin collector is loaded and does not limit expansion much. Therefore, the curvature gradually decreases with an increasing thickness ratio.

**Figure 5:**
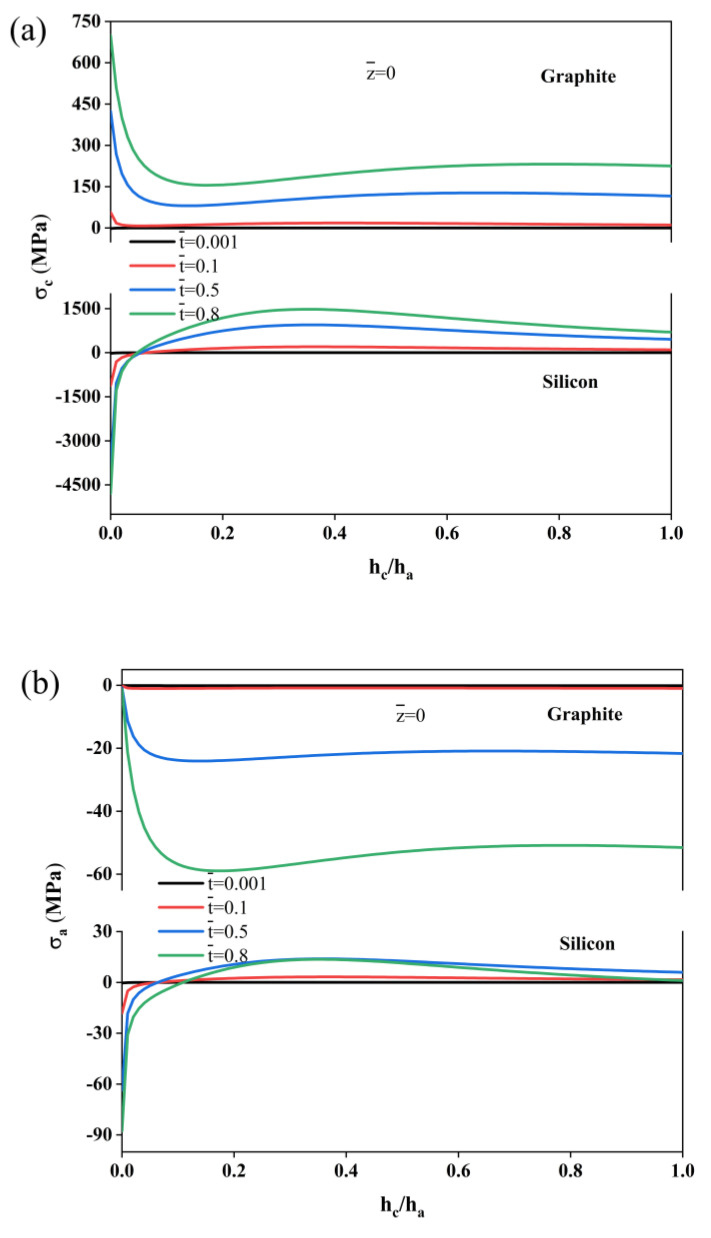

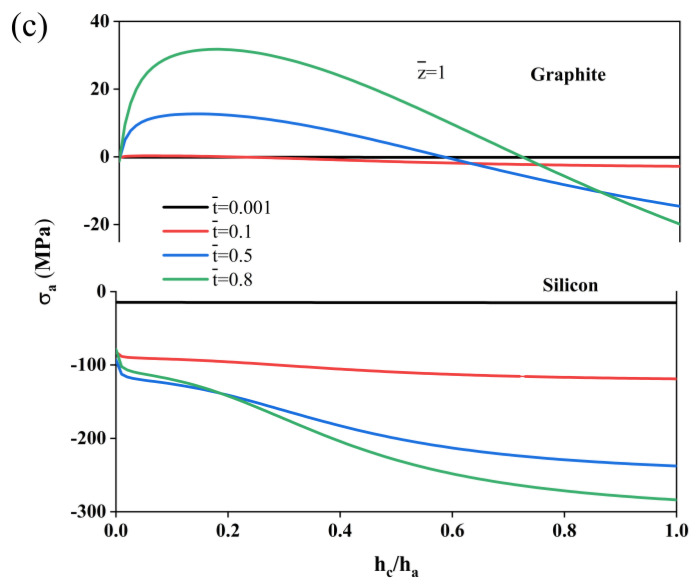
Simulated stress trends of single-layer composite electrodes with different thickness ratios. (**a**) Collector interface stress, (**b**) stress of the active layer interface and (**c**) surface stress of the active layer.

Figure 5a illustrates the stress at the interface of the current collector during the charging process. For both the graphite and silicon composite electrodes, when $h_{c}/h_{a}$ is not very large, the stress decreases sharply with increasing collector thickness. However, for the graphite composite electrode, the stress decreases are from the tensile stress, and the stress increases again when the $h_{c}$/$h_{a}$ ratio reaches 0.1. This is due to the decrease in the curvature of the electrode, which causes the transformation of the stress of the current collector from bending to tension. Consequently, the stress rises slightly. In contrast, for the silicon composite electrode, the stress decreases sharply due to the compressive stress, and changes to tensile stress after $h_{c}/h_{a}=0.1$. Figure 5b,c show the change in the stresses in the active layer for different thickness ratios at both interfaces (z = 0 and z = 1) throughout the charging process. It can be observed that an increase in the thickness ratio results in a rise in the peak stress, regardless of whether it is tensile or compressive. Furthermore, thicker collectors result in greater constraint, leading to more stress and a corresponding reduction in the durability of both the graphite and silicon composite electrodes. To minimize the occurrence of stress peaks and improve the efficiency of the composite electrodes, graphite composite electrodes should select a current collector with the smallest possible thickness ratio, while silicon composite electrodes should select a collector with a thickness ratio of around 0.1.

## 3. Experimental Section

### 3.1. Electrode Preparation

**Figure 6:**
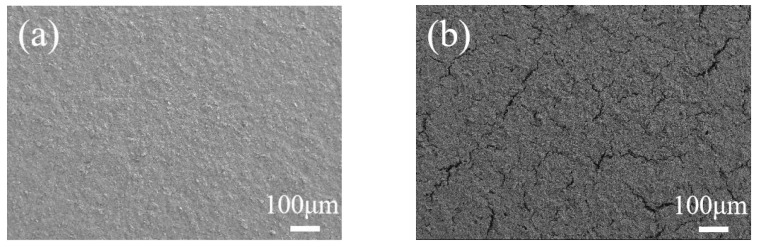
Surface microstructure of the composite electrodes captured by SEM. (**a**) Graphite electrode and (**b**) silicon electrode.

In this work, the composite electrodes were made by mixing the active particle, carbon black and polymeric binder to form the uniform slurry. De-ionized water was used as the solvent, while copper foil worked as the current collector. Here, the mass ratio of each component was 8:1:1 for the graphite electrode, while it was 2:1:1 for the silicon composite electrode, respectively. Subsequently, the prepared electrodes were first left for one night at room temperature in the coating machine; then, the electrodes were dried at 85 °C for 6 h under a vacuum state. The surface morphology is shown in Figure 6. The mass loading was measured as 6.54 mg cm^−1^ and 3.18 mg cm^−1^ for the graphite and silicon composite electrodes, respectively.

### 3.2. Assembly of the Model Cell

**Figure 7:**
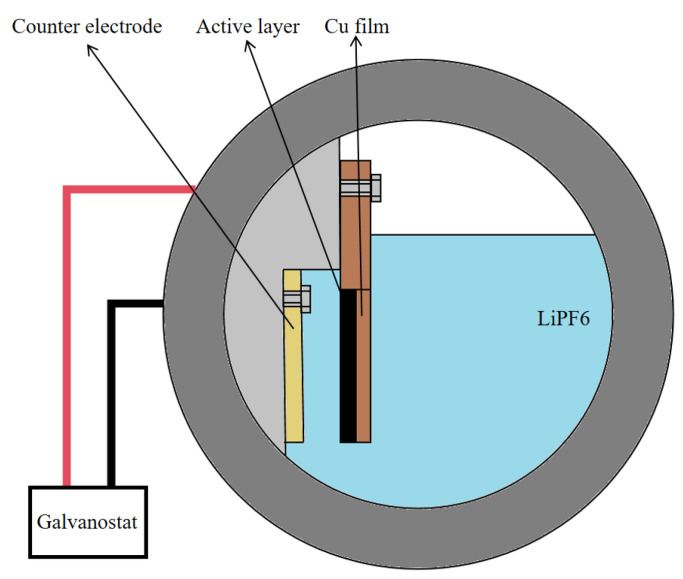
Structure of model battery.

Here, a model cell was developed to analyze the mechanical response during electrochemical cycling. It was assembled in a glove box with an ultra-pure argon environment (Mikrouna), and its structure is shown in Figure 7. Here, LiFePO4 electrodes were used as the reference electrodes to supply sufficient lithium ions, while the composite electrodes were used as the working electrodes. A microporous polypropylene film (Celgard 2400, 25 μm) was used as a separator to prevent the short circuiting of the electrodes. The working electrodes and counter electrodes were arranged in a cantilevered configuration (the active level of the working electrodes was oriented towards the counter electrodes) to facilitate lithium ion transport. Then, all the components of the model cell were fully immersed in an electrolyte (1 M LiPF6, EC: DEC = 1:1 V). The model cell was assembled using a sealing gasket and a transparent quartz cap, with the upper-end cap, transparent quartz cap and base of the model cell fixed using fastening nuts. After the cell was prepared, it was held in a glove box for 3 h. This process helped to ensure that the electrodes were completely infiltrated by the electrolyte.

### 3.3. Electrochemical Measurements

When the cell was fully immersed in the electrolyte, a constant current was applied to conduct electrochemical cycling by a battery tester (NEWARE) at a voltage of 2.0–4.2 V. After each lithiation and delithiation process, an open-circuit relaxation period of 60 min was initiated. During this period, the lithiation/delithiation time employed during the test was used to control the state of charge of the battery. Then, the deformation of the composite electrodes was performed by the CCD camera through the transparent quartz in the model cell, every two minutes during the electrochemical cycle. Meanwhile, the electrochemical workstation recorded the electrochemical data, i.e., the voltage, current and time. The curvature of the composite electrodes was obtained by using AutoCAD for the subsequent electro-mechanical coupling analysis.

## 4. Modeling of the Bending Deformation of the Composite Electrodes

**Figure 8:**
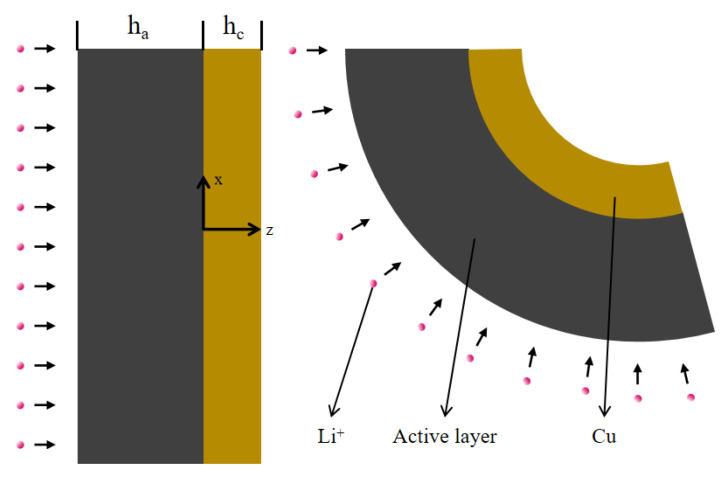
Diagram of composite electrode.

Combining the simulations with the experimental results, a physical model was built, as shown in Figure 8. The structure of the electrode consists of two sections, the active layer and the current collector. The thickness of the active layer is $h_{a}$ and $h_{c}$ is the thickness of the copper foil. The active layer can expand, which is caused by the insertion of Li ions, and, at the same time, the current collector restricts its volume change. This, in turn, causes the electrode to bend. In the mathematical model, the *z*-axis is aligned with the thickness of the electrode, while the *x*-axis and *y*-axis are located at the interface of the two different layers. The collector and the active layer exhibit the same bending deformation during the lithiation process. For the purpose of modeling feasibility, it is assumed that the coefficients remain constant and the porosity of the active layer is uniform. According to these assumptions, the lithium ion diffusion equation of the laminated electrode structure is as follows:

(1) $$\frac{\partial c}{\partial t}-D\frac{\partial^{2}c}{\partial z^{2}}=0$$

where D is the effective diffusivity of the lithium ions and c is the concentration of lithium ions in the active layer, which are two key factors for lithium ion diffusion. Assuming that initially there are no lithium ions in the composite electrodes, a constant current boundary condition with uniform current density is provided on the outer surface of the active layer ($z=h_{a}$).

(2a) $$c\left(z,t\right)=0,\;for\;t=0$$

(2b) $$D\frac{\partial c(z,t)}{\partial z}=\frac{I_{0}}{F},\;for\;z=h_{a}$$

(2c) $$D\frac{\partial c(z,t)}{\partial z}=0,\;for\;z=0$$

The quantity $I_{0}$ represents the current density of the outer surface of the active layer and $F=96,485.3\;C\;{\mathrm{mol}}^{-1}$ denotes the Faraday constant. It is important to note that the boundary conditions specified in Equations (2a)–(2c) apply to larger currents in layered electrodes. Consequently, the solution to the above margin problem for the lithium ion concentration along the *z*-axis can be expressed as follows:

(3) $$c\left(z,t\right)=\frac{I_{0}h_{a}}{FD}\{\frac{Dt}{{h_{a}}^{2}}+\frac{3z^{2}-{h_{a}}^{2}}{6{h_{a}}^{2}}-\frac{2}{\pi^{2}}\sum_{m=1}^{\infty }\frac{{\left(-1\right)}^{m}}{m^{2}}\times \cos\left(\frac{m\pi z}{h_{a}}\right)exp(-\frac{m^{2}\pi^{2}Dt}{{h_{a}}^{2}})\}$$

Here, the electrodes are assumed to be macroscopically elastic, homogeneous and isotropic [9]. The Li ion concentration has a significant influence on the mechanical response of the electrodes. Under the assumption of a small deformation, it can be assumed that ε, the strain in plain, varies linearly along the *z*-axis.

(4) $$\varepsilon =\varepsilon_{0}+\kappa z$$

where $\varepsilon_{0}\;$is defined as the in-plane strain at the interface (z = 0) and κ denotes the curvature of the battery electrode. In addition, the stress within the active layer can be assumed to be as follows [16]:

(5a) $$\sigma_{a}=E_{a}\left(\varepsilon_{0}+\kappa z\right)-E_{a}\Omega c/3$$

where Ω is the partial molar volume and $\Omega_{c}$ denotes the expansion strain of the active layer in the saturated state. Since the copper foil is not affected by lithium ions, the stress in the collector can be obtained from the following equation [16]:

(5b) $$\sigma_{c}=E_{c}\left(\varepsilon_{0}+\kappa z\right)$$

where $E_{c}$ is the modulus of the collector and the elastic modulus of the collector is 117 GPa. In this instance, the electrodes are at the traction-free boundary, as shown below.

(6) $$\int_{z}\sigma_{x}dz=0,\int_{z}\sigma_{x}zdz=0$$

The substitution of Equation (5) into Equation (6) gives the in-plane strain and bending curvature of the battery electrode, respectively, as follows:

(7) $$\varepsilon_{0}=\frac{IN^{c}-BM^{c}}{AI-B^{2}}$$

(8) $$\kappa =\frac{AM^{c}-BN^{c}}{AI-B^{2}}$$

(9) $$\begin{array}{c} A=\int_{z}Edz,\;B=\int_{z}Ezdz,\;I=\int_{z}Ez^{2}dz,\;\\ N^{c}=\frac{1}{3}\int_{z}E\Omega cdz,\;M^{c}=\frac{1}{3}\int_{z}E\Omega czdz \end{array}$$

The effect of the elastic modulus, which is influenced by the concentration of lithium ions, on the mechanical response of the active layer has been considered, due to the higher charging rate. Other researchers have obtained electrode moduli through nanoindentation that satisfies a linear distribution, employing a simple primary function to investigate the influence of elastic modulus changes on stress levels in response to the lithium ion concentration [28]. Therefore, $E_{a},$ the elastic modulus of the active layer, should obey a linear function.

(10) $$E_{a}=E_{a0}+\lambda \bar{c}$$

Considering the large volume expansion of the particular volume and the uniform distribution and isotropy of the active substance, the partial molar volume of the electrode can be calculated using the following equation [29]:

(11) $$\Omega =\Omega_{0}+\beta \bar{c}$$

where $\Omega_{0}$ is the initial partial molar volume of the electrode and is assumed to be a constant as the particles expand during lithiation, which is shown in Table 1.

$E_{a0}$ denotes the initial modulus for the active layer. λ is a negative constant for the silicon electrode because of its softening property during the lithiation process [30]. In contrast, it is a positive value for the graphite electrode. The above constants are shown in Table 1. $\bar{c}=c/c_{s}$ is defined as the dimensionless lithium ion concentration, where $c_{s}$ is the maximum lithium ion concentration at saturation.

By substituting Equations (9)–(11) and the initial material parameters of the composite electrodes from Table 1 into Equations (7) and (8), the curvature and in-plane strain equations for the graphite and silicon composite electrodes can be calculated, respectively. Further derivations can obtain the stress equations for the current collector and bilayer, along with the electrode thickness.

The analytical model described above allows for the stress distribution in the laminated electrodes to be determined at different charging multiplicities. Considering the nonlinear effect of the lithium ion concentration on the stress solution, a computational analysis was conducted using COMSOL Multiphysics@.

## 5. Conclusions

We experimentally measured the bending of the composite electrodes with an increasing C-rate, due to the asymmetric diffusion of Li ions in the composite electrodes and the limitations of the copper foil. Based on the experiments, a model of composite electrodes was developed, and the simulated deformation results fitted consistently with the experimental results. It was also found that the charging multiplicity and thickness ratio have a significant effect on the mechanical parameters of the composite electrodes. For the silicon composite electrode, the curvature increases with an increase in the multiplicity. For the graphite composite electrode, the curvature decreases with an increase in the magnification, due to graphite hardening. And an increase in the thickness ratio causes an increase in the peak stress for both composite electrodes.

## Figures and Tables

**Table 1:** Initial material parameters of composite electrodes for computational modeling.

	Silicon	Graphite
$E_{a}$	1.9 (Gpa)	0.5 (Gpa)
λ	−0.17	15
Ω	$1.21\times 10^{-6}(m^{3}/mol)$	$1.47\times 10^{-6}\;(m^{3}/mol)$
β	$5\times 10^{-8}$	0

## Data Availability

No new data were created.
